# Comparison of Laboratory Confirmed Drugs in Acute Recreational Drug Toxicity Presentations to an Urban Hospital in London, UK, 2016/17 versus 2019/20

**DOI:** 10.1007/s13181-024-01051-8

**Published:** 2024-12-11

**Authors:** Caitlin E Wolfe, Lachlan J Sund, John RH Archer, Ashley Rowe, Simon Hudson, David M Wood, Paul I Dargan

**Affiliations:** 1 Atlantic Canada Poison Centre, Nova Scotia, Canada; 2 Emergency Medicine, Guy’s and St Thomas’ NHS Foundation Trust and King’s Health Partners, London, UK; 3 Clinical Toxicology, Guy’s and St Thomas’ NHS Foundation Trust and King’s Health Partners, London, UK; 4 Faculty of Life Sciences and Medicine, King’s College London, London, UK; 5 Health Canada, Ottawa, Canada; 6 Sport and Specialised Analytical Services, LGC Assure Ltd, Fordham, UK

**Keywords:** NPS, Novel psychoactive substances, Legal highs, Recreational drugs

## Abstract

**Introduction:**

Novel Psychoactive Substance (NPS) use is increasingly prevalent and is often associated with severe acute recreational drug toxicity (ARDT). 258 UK deaths were attributed to NPS use in 2021. Confirmatory testing which identifies NPS is limited by expense and timeliness. We aimed to identify NPS and other recreational drugs in a sample of 1000 ARDT presentations to a central London hospital in 2019/20 and to compare these drugs to those identified from a previous cohort in 2016/2017.

**Methods:**

We prospectively enrolled 1000 serum samples from ARDT presentations to St Thomas’ Hospital between February 2019 and February 2020. Serum samples were deidentified and underwent qualitative analysis via mass spectrometry. Results were returned at the conclusion of testing and statistical analysis performed using ‘R’ (R Foundation for Statistical Computing).

**Results:**

Twenty-eight unique NPS were detected in 2019/20, compared to 31 in 2016/17. Eight new NPS were detected in 2019/20: four benzodiazepines, two synthetic cannabinoid receptor agonists, one cathinone and one ketamine-analogue. No NPS opioids were detected in either cohort. Cannabis (16%,11% *p* = 0.02), ketamine (12%,7% *p* < 0.01) and opioids (57%,24% *p* < 0.01) were detected significantly more frequently in 2019/20 than in 2016/17, while alcohol (22%,49% *p* < 0.01), cathinones (1%,15% *p* < 0.01), GHB (14%,20% *p* < 0.01) and MDMA (9%,18% *p* < 0.01) were detected less frequently.

**Conclusions:**

Studies that utilise confirmatory testing to detect NPS in presentations of ARDT provide important information for public health interventions. More NPS benzodiazepines and fewer NPS cathinones were detected in 2019/20, following temporal trends of forensic detection throughout Europe and reinforcing the importance of identifying emerging drugs.

**Supplementary Information:**

The online version contains supplementary material available at 10.1007/s13181-024-01051-8.

## Introduction

Acute recreational drug toxicity (ARDT) remains a common presentation to United Kingdom (UK) hospitals, with 16,994 hospital admissions in England alone throughout 2019/20 [1]. Both locally and internationally there has been a significant change in the drugs used over the last 15 years. Novel psychoactive substances (NPS), also referred to as ‘legal highs’, have become increasingly available with tens of new substances of different drug classes being detected yearly [2]. Owing to their rapid emergence, there is limited population level data on the prevalence of NPS use and limited information on the clinical effects of specific substances. The European Drug Emergencies Network Plus (Euro-DEN Plus), who monitor presentations of ARDT to emergency departments across Europe, reported that 7.6% of presentations to participating centres involved at least one NPS between 2014 and 2019 [3]. NPS are also increasingly being associated with fatalities: in 2021 258 deaths were attributed to NPS use throughout England and Wales, a sharp rise from the five deaths attributed to NPS in 2005 [4].

At an individual level most presentations of recreational drug and NPS toxicity are clinically managed according to self-report of drugs used as confirmatory testing and analysis is often not available in a timely manner and can be expensive to undertake. NPS are generally designed to mimic the effects of controlled substances and it is likely that NPS remain undetected at the individual level in many cases [5–7]. Consequently, confirmatory testing plays a crucial role in identifying emerging trends and raising public-health issues relating to cases of severe toxicity. An early example of this was the STRIDA project which identified 159 NPS from selected ARDT presentations to Swedish emergency departments and intensive care units between 2010 and 2016 [8]. Users may also be unknowingly exposed to NPS, as was the case for the novel opioids of the nitazene class; these potent synthetic opioids have been responsible for clusters of opioid-related deaths and severe poisonings in heroin users in the UK since 2021 [9–11].

Our research team had previously undertaken confirmatory testing on serum samples from 500 ARDT presentations prospectively identified to our inner city London hospital between October 2016 and February 2017; NPS belonging to the following classes were detected: benzodiazepines, cathinones, synthetic cannabinoid receptor agonists (SCRA), phenylethylamines and phenylpiperazines [12]. The aim of this study was to report on the NPS detected in a second cohort of 1,000 samples from ARDT presentations to the same hospital in 2019/2020 in order to determine changes in the NPS and recreational drugs involved in ARDT presentations.

## Methods

Cases were enrolled prospectively from a convenience sample of 1,000 adult (18 years or older) ARDT presentations to the emergency department of a large, tertiary, inner city hospital in London, UK, between 1st February 2019 and 24th February 2020. Preliminary chart review was undertaken for relevant discharge diagnoses from the emergency department within the previous 24–72 h. Cases were excluded if a serum sample had not been collected during initial assessment or if the presentation was suspected to be due to lone alcohol use/intoxication. De-identified demographic and clinical data were collected in Microsoft Excel.

Serum samples were collected from the biochemistry department of the on-site laboratory once routine analysis had been performed providing sufficient volume remained for confirmatory analysis (more than 0.5mL). Each sample was then coded and placed into a new tube with a de-identified case number. The serum samples were frozen and sent in batches to an independent laboratory (LGC Assure Ltd.– Sport and Specialised Analytical Services) for analysis. Qualitative analysis was conducted using high resolution mass spectrometry with comparison to a database of known drugs and metabolites. This database continues to be updated at least weekly from a number of catalogues including the European Union Drug Agency (EUDA) Early Warning System (EWS), the UK Forensic EWS, the US Center for Forensic Science Research and Education NPS discovery program, and the international ‘HighResNPS’ catalogue. When substances are added, the mass of expected metabolites and/or experimentally determined metabolites are also added to the database. In this instance, the testing laboratory was blinded to clinical presentation, patient demographics, or suspected toxicity. Any metabolite that was detected was coded for the purposes of this study as a positive result for its parent compound.

Results were returned to the research team at the conclusion of testing and statistical analysis was performed using R (R Foundation for Statistical Computing). Pearson’s chi-squared test at 5% significance level was used to compare patient gender and the prevalence of use of specific drug classes between the two cohorts. Student’s t-test at 5% significance was used to compare the mean age between the two cohorts.

Ethical Approval for the study was granted by the research and ethics committee of the Yorkshire and the Humber– Leeds East, Health and Research Authority of the National Health Service (REC reference 14/YH/N040); it was determined that individual consent for testing of serum samples was not required.

## Results

Of the 1,000 samples in cohort 2, a total of 939 were suitable for analysis and 61 were excluded as follows: (i) 49 samples contained insufficient volume for analysis; (ii) six had no report or clinical suspicion of recreational drug use; (iii) three had no recorded age; (iv) two were from individuals younger than 18 years; (v) one was a lone alcohol ingestion. Males were involved in 85% of the cases, consistent with the previous cohort of 500 samples, which was also 85% male. The mean age of 34.8 years in cohort 2 (median age 42, IQR 27–42) was marginally older than the 33.2 years of cohort 1 (median age 32 IQR 26–40) (*p* < 0.01).

The NPS detected in both cohorts are listed in Table 1. A number of substances were common to both cohorts as shown in the middle column. A total of 39 unique NPS were detected between the cohorts with 20 of those detected in both cohorts, 11 in cohort 1 only and eight in cohort 2 only. The classes with the highest number of unique NPS detected throughout either cohort were cathinones (16), SCRA (11) and benzodiazepines (seven).

Four new NPS of the benzodiazepine class were detected in cohort 2 in comparison to the one benzodiazepine unique to cohort 1. Conversely, only one new synthetic cathinone was detected in cohort 2 in comparison to the six cathinones unique to cohort 1. Two new SCRA were detected in cohort 2 in comparison to the three SCRA unique to cohort 1. Cohort 2 was also the first time a ketamine-analogue had been detected (deschloro-n-ethylketamine). No NPS opioids were detected in either cohort.

**Table 1:** NPS detected in cohort 1 (2016/17) and cohort 2 (2019/20)

NPS class	Unique to cohort 1 (2016/17)	Detected in both	Unique to cohort 2 (2019/20)
Benzodiazepine	Flubromazepam	Diclazepam Etizolam	Clonazolam Flualprazolam Flubromazolam Fluclotizolam
Cathinone	Alpha-PVP* Chloro-n-ethylcathinone Chloro-PPP* Dibutylone Ethylone Mexedrone	4-chloro-ethcathinone 4-methyl-pentedrone 4-ethyl-ethcathinone Mephedrone Methylone 4-MEAP* Clephedrone Ephylone Metamfepramone	4-methyl-N, N-diethylcathinone
Ketamine analogue			Deschloro-n-ethylketamine
Synthetic cannabinoid receptor agonist	Cumyl-5 F PINACA AB-CHMINACA AB-FUBINACA	5 F ADB 5 F AKB-48 AMB-CHMICA BB-22 MDMB-4en-PINACA MDMB-CHMICA	4 F MDMB-BINACA 5 F MDMB-PICA
Phenylethylamine	Methiopropamine	4 F-MPH* DOC	
Phenylpiperazine		m-CPP*	

*Alpha-PVP = alpha-pyrrolidinopentiophenone; Chloro-PPP = chloro-pyrrolidinopropiophenone; 4-MEAP = 4-methyl-alpha-ethylaminopentiophenone; 4 F-MPH = 4-fluoromethylphenidate; DOC = 2,5-dimethoxy-4-chloroamphetamine; m-CPP = meta-chlorophenylpiperazine

Table 2 demonstrates the frequency of detection of each drug class across the two cohorts, both NPS and established recreational drugs. Cannabis, ketamine and opioids were detected significantly more frequently in 2019/20 than in 2016/17. In comparison, alcohol, cathinones, GHB (gamma-hydroxybutyrate), and MDMA (3,4-methylenedioxymethamphetamine) were detected significantly less frequently in 2019/20. There was no significant difference in the frequency of detection of amphetamine, benzodiazepines, cocaine, methamphetamine and SCRA between the two cohorts.

**Table 2:** Frequency of detection of each substance/drug class in 2016/17 and 2019/20

Substance/drug class^	2016/17 cohort 1 (%) [*n* = 500]	2019/20 cohort 2 (%) [*n* = 939]	*p*-value*
Alcohol	244 (49%)	202 (22%)	**< 0.01**
Amphetamine	100 (20%)	166 (18%)	0.28
Benzodiazepine	239 (44%)	496 (53%)	0.07
Cannabis	56 (11%)	147 (16%)	**0.02**
Cathinones	76 (15%)	13 (1%)	**< 0.01**
Cocaine	190 (38%)	347 (37%)	0.70
GHB	102 (20%)	136 (14%)	**< 0.01**
Ketamine	36 (7%)	110 (12%)	**< 0.01**
MDMA	89 (18%)	85 (9%)	**< 0.01**
Methamphetamine	119 (24%)	245 (26%)	0.34
Opioid	122 (24%)	534 (57%)	**< 0.01**
Synthetic cannabinoid	74 (15%)	149 (16%)	0.59

^ Substance/drug classes with > 5 detections

* 5% (0.05) significance level

## Discussion

The detection of eight distinct new NPS in 2019/20 compared to 2016/17 highlights the rapidly evolving nature of the NPS market and its impact on patterns of recreational drug use and associated harms in London. Four (50%) of these substances were of the benzodiazepine class, two (25%) were of the SCRA class and one (12.5%) was a cathinone. Evolution of the market is further evidenced by the 11 NPS that were detected in 2016/17 but not again in 2019/20, likely representative of a reduction in the circulation of these drugs. The EUDA (previously the European Monitoring Centre for Drugs and Drug Addiction) EWS, which identifies and monitors NPS throughout Europe (via a combination of drug seizures, blood samples, autopsies), identified 16 new NPS of the benzodiazepine class in the years 2016–2019 [2]. It is likely that cohort 2 within this study is representative of the increase in the availability of these drugs throughout Europe. A similar trend can be observed for cathinones– the EUDA EWS detected for the first time 69 NPS of the cathinone class in the years 2012–2015 compared to 44 between 2016 and 2019. This corresponds temporally with the higher number of new cathinones detected in cohort 1 of this study. SCRA continue to be detected in large numbers by the EUDA EWS, with 154 NPS detected for the first time between 2012 and 2019. The Euro-DEN Plus group also reported that a majority of NPS presentations were due to SCRA between 2016 and 2019 (ranging between 49.9% and 72.2%) and that the proportion attributable to synthetic cathinones had fallen (33.0% in 2016, 11.6% in 2019) [3]. 2019 was the first year of Euro-DEN Plus reporting since 2015 that the ‘other NPS’ category, which includes novel benzodiazepines and opioids (in addition to other classes), had the second highest number of agents reported.

It should be noted that the EWS reported 41 NPS opioids in Europe for the first time between 2016 and 2019, however no synthetic opioids were detected in either cohort. This was despite opioids being detected in a significant proportion of presentations (24% in 2016/17 and 57% in 2019/20). The subsequent cluster of opioid-related deaths and severe poisonings within the UK from August 2021 onwards (16 months after the final sample of cohort 2 within this study) may be suggestive of a relationship between EWS detection and delayed emergency presentations of acute drug toxicity. These deaths also validate the importance of regular surveillance of ED ARDT presentations to determine the NPS responsible and to inform decisions regarding drug control and harm minimisation.

Patterns of overall drug use locally changed between 2016/17 and 2019/20, as evidenced by the changes in the detection rates of each drug class between the two cohorts. In 2019/20 there was a considerably higher opioid detection rate (*p* < 0.01), as well as more cannabis (*p* = 0.02) and ketamine detection (*p* < 0.01). There was also less frequent alcohol, cathinone and GHB detection (*p* < 0.01). Concerningly the increase in opioid detection corresponded with an increase in opioid-related deaths in the UK from 35.7 per million in 2016 up to 39.1 per million in 2020 [4]. An increase in opioid usage may also be predictive of an increase in future NPS opioid detection, as manufacturers innovate in response to demand and regulatory pressures [13]. An example of such regulatory pressures emerged in Afghanistan, previously the worlds largest producer of heroin, where local authorities prohibited opium poppy cultivation in April 2022 and production subsequently fell by 95% in 2023 [14]. The STRIDA project in Sweden also demonstrated the disappearance from market of a number of NPS post identification as harmful substances [8].

Using excess serum samples obviates some of the potential bias and under-reporting of usage surveys however, it should be acknowledged that this approach cannot be used to assess population level use. Substances resulting in moderate-severe toxicity are likely to be overrepresented in the ED and thus our detection rates are less reflective of overall population-level use. Another limitation of this study was that serum samples were only taken when biochemical testing was deemed necessary on the clinical judgement of the attending emergency clinician. It is therefore difficult to comment on the absolute prevalence of NPS resulting in ARDT presentations however, it should be noted that the majority of patients have blood samples sent during an ED attendance. Given urine samples are less frequently collected in the emergency department, they were not utilised in this study.

The number of UK adults reporting lifetime use of cannabis and ketamine has been rising since 2016, consistent with the findings of our study [15]. Similarly, self-reported lifetime use of ‘mephedrone’, an NPS cathinone, has been falling over the same time period. Despite detection rates of MDMA falling between 2016/17 and 2019/20 in this study, self-reported lifetime use of ‘ecstasy’ remained stable between nine and 10% over the same time [15].

## Conclusions

This study detected the presence of 28 NPS in blood samples from a cohort of ARDT presentations to a central London Hospital in 2019/20. Eight of these NPS had not been detected previously. Conversely, 11 of the NPS detected in 2016/17 were not detected again in 2019/20. More NPS benzodiazepines and fewer NPS cathinones were detected in 2019/20, following temporal trends of forensic detection throughout Europe and reinforcing the importance of the EWS in identifying emerging drugs and issuing public health warnings.

## Electronic Supplementary Material

Below is the link to the electronic supplementary material.

Supplementary Material 1
